# Supplementary material for: CD8+ T cells drive myofibroblast activation and contraction via JAK/STAT3 and TGFβ signaling
Source: iScience. 2025 Oct 30;28(12):113904. doi: 10.1016/j.isci.2025.113904 (PMC12664390; doi:10.1016/j.isci.2025.113904)
Supplement: Document S1. Figures S1–S8 and Tables S1–S4 [file mmc1.pdf]

iScience, Volume *28*

Supplemental information

CD8+ T cells drive myofibroblast activation and contraction via JAK/STAT3 and TGFβ signaling

Theodoros Ioannis Papadimitriou, Anne van Essen, Merel Willemse, Daphne Dorst, Elly Vitters, Birgitte Walgreen, Hans J.P.M. Koenen, Peter van der Kraan, Marije Koenders, Rogier M. Thurlings, and Arjan van Caam

Supplemental Figures

Supplemental figure 1

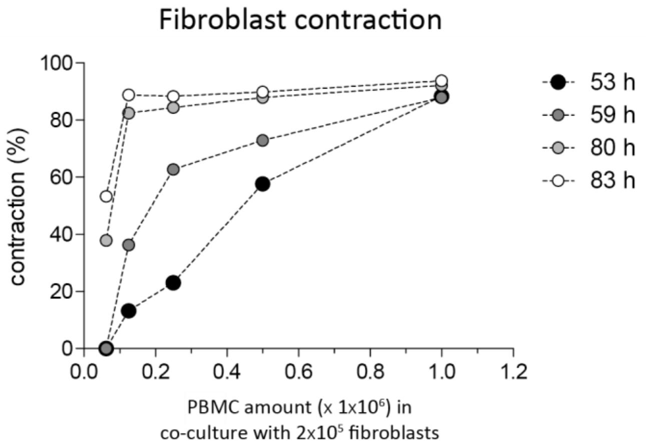

Supplemental figure 1. Contraction of hydrogels based on time and PBMC: myofibroblast ratio. Primary skin myofibroblasts were co-cultured with varying amounts of PBMCs in collagen type 1 hydrogels. The extent of hydrogel contraction was measured at specific time points as indicated.

PBMCs; Peripheral Blood Mononuclear Cells

Supplemental figure 2

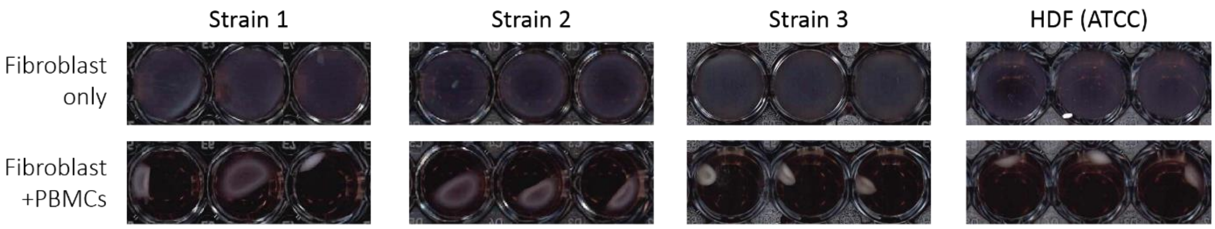

Supplemental figure 2. PBMC-myofibroblast co-culture induced hydrogel contraction in different myofibroblast strains. Four strains of primary skin myofibroblasts show strong contraction after 72 h of co-cultured with PBMCs in collagen type 1 hydrogels. This includes a primary normal dermal myofibroblasts (HDF) obtained from ATCC.

PBMCs; Peripheral Blood Mononuclear Cells, HDF; Human Dermal Myofibroblasts, ATCC; American Type Culture Collection.

Supplemental figure 3

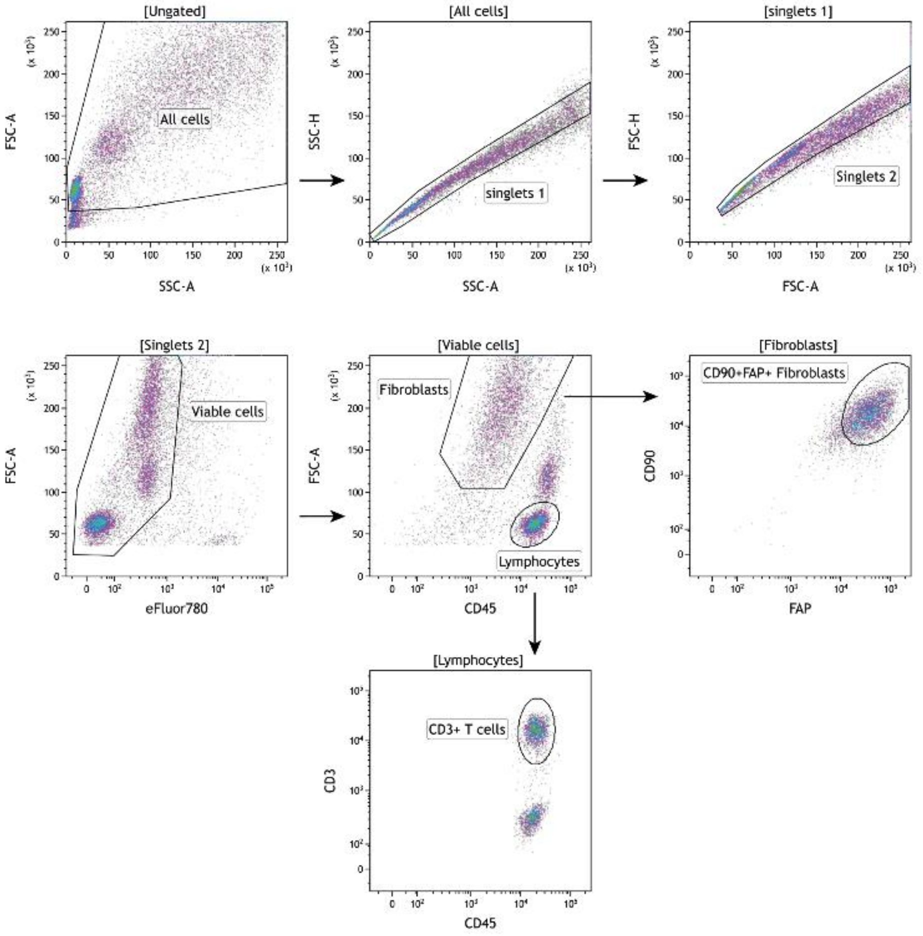

Supplemental figure 3. FACs gating strategy for sorting Myofibroblast and T cells for RNA analysis. Myofibroblasts were defined as $CD45^-$, and $CD90^+$, FAP^+.

FACs; Fluorescence-Activated Cell sorting.

Supplemental figure 4

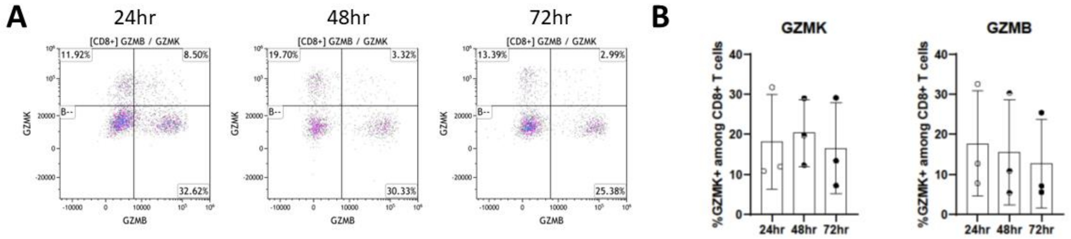

Supplemental figure 4. PBMC-myofibroblast co-culture does not affect the percentage of CD8+GZMB+ nor CD8+GZMK+ T cells. (A) Flow cytometry plots from one representative donor exhibit expression of GZMB and GZMK in CD8+ T cells co-cultured with myofibroblasts for 24, 48 and 72 hours. (B) Graphic quantification of the percentage of CD8+GZMB+ and CD8+GZMK+ T cells from n=3 donors at 24, 48 and 72 hours of co-culture.

PBMCs; Peripheral Blood Mononuclear Cells, GZMK; Granzyme K, GZMB; Granzyme B.

Supplemental figure 5

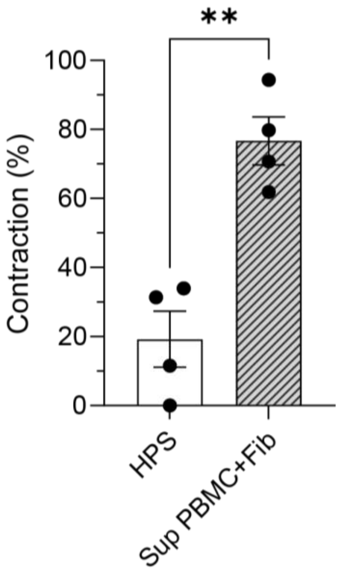

Supplemental figure 5. Co-culture supernatant transfer promotes hydrogel contraction in myofibroblast monoculture. Myofibroblasts and PBMCs were co-cultured for 72 hours, after which the supernatant was collected and centrifuged twice at 300 x g. This processed supernatant (20% v/v) was then added to the medium of fresh hydrogels containing only myofibroblasts. As a control, medium that had not been exposed to co-cultures (10% HPS) was used. The supernatant-induced contraction of myofibroblast monocultures was observed 1-2 days later compared to when immune cells are present.

PBMCs; Peripheral Blood Mononuclear Cells, HPS; Human Pooled Serum.

Supplemental figure 6

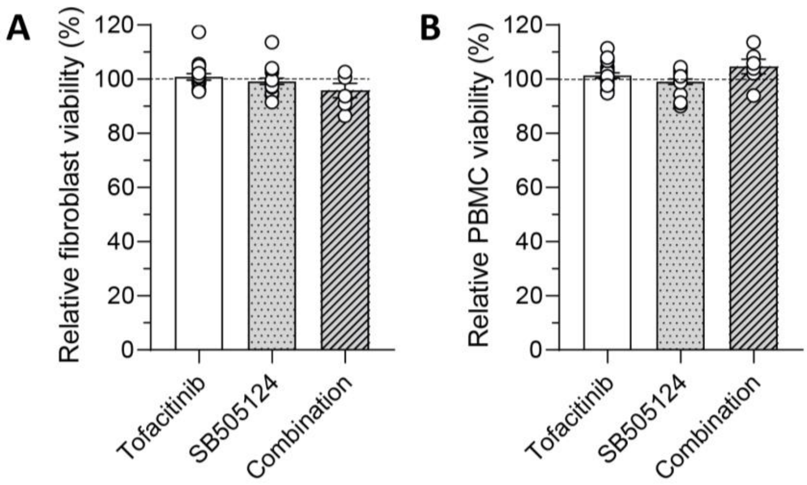

Supplemental figure 6. No significant decrease in myofibroblast nor PBMC viability is observed with the inhibitors' treatment. Relative viability of myofibroblasts (A) and PBMCs (B) after 72-hour treatment with Tofacitinib (0.5 µM), SB505124 (5 µM) or the combination of both compared to vehicle control.

Supplemental figure 7

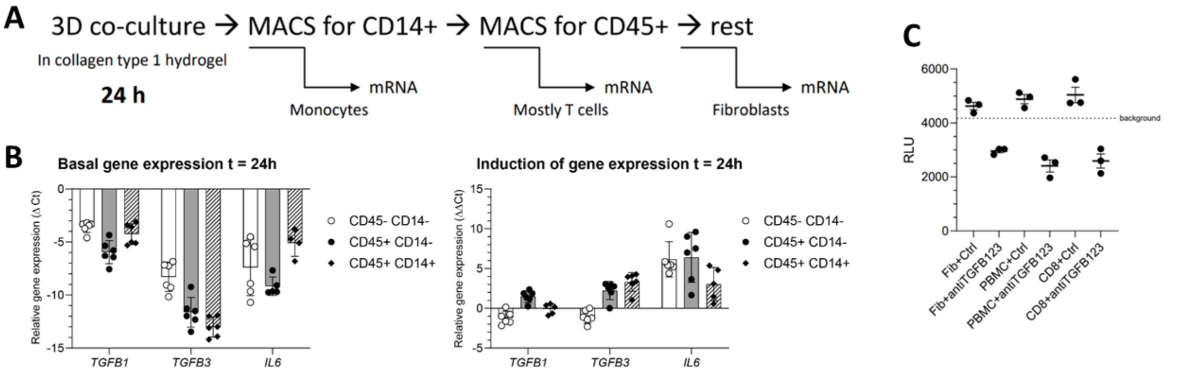

Supplemental figure 7. TGFβ signaling plays an essential role in immune-cell mediated myofibroblast contraction and activation. A) Schematic representation of the sorting strategy that was used to isolate fibroblasts (CD45-CD14-), monocytes/macrophages (CD45+CD14+) and CD45+CD14- immune cells (containing lymphocytes of which the largest amount is T cells) for RNA isolation 24 hours after co-culture. B) Basal gene expression (left) and induction of gene expression (right) of TGFB1, TGFB3 and IL6 in the sorted myofibroblasts, monocytes/macrophages and lymphocytes. Values are represented as relative gene expression (-ΔCt) III) RLU luciferase values of SBE expression in SBE reporter cells that were cultured with the designated supernatants and were treated with isotype (control) or anti-$TGFB_{123}$ antibody for 1 hour at 37 degrees Celcius.

Supplemental figure 8

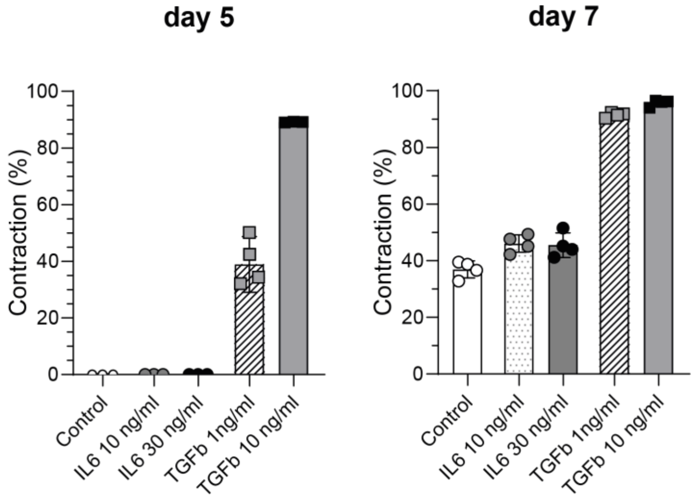

Supplemental figure 8. Spontaneous contraction of myofibroblast monoculture hydrogels under the influence of TGFB and IL6. Primary skin myofibroblasts were cultured in collagen hydrogels for 1 week and contraction was measured at day 5 and day 7. At day 5, no spontaneous contraction is observed in the controls yet, whereas at day 7 this can be observed. TGFβ clearly enhanced contraction, but IL-6 alone did not.

Supplementary materials

Supplementary Table 1: List of antibodies used for immunohistochemistry

Antigen	Clone	Supplier	Identifier
Pro-collagen type 1	Polyclonal	Merck	ABT257
alpha-smooth muscle actin	Polyclonal	Abcam	ab5694
Podoplanin	D2-40	Biolegend	916605
Fibroblast activation protein	EPR20021	Abcam	ab207178
Phospho-SMAD2 (Ser465/467)	138D4	CellSignaling	#3108
Phospho-Stat3 (Tyr705)	D3A7	CellSignaling	9145S
Cleaved caspase-3 (Asp175)	Polyclonal	CellSignaling	9661S
γH2AX	Polyclonal	CellSignaling	9718S

Supplementary Table 2: List of antibodies used for cell surface flow cytometry staining

Antigen	Clone	Fluorochrome	Supplier	Identifier
CD4	RPA-T4	PerCP/Cy5.5	Biolegend	300530
CD3	UCHT1	Alexa Fluor700	Biolegend	300425
CD8a	RPA-T8	BV510	Biolegend	301048
CD45	HI30	BV510	Biolegend	304036
CD25	M-A251	PE/Cy7	Biolegend	356108
CD134 (OX40)	ACT35	FITC	Biolegend	350006
HLA-DR	L243	APC	Biolegend	307609
HLA-A,B,C	W6/32	APC	Biolegend	311409
CD90 (Thy-1)	5E10	APC	eBioscience	17-0909
FAP	BLR150J	APC	R&D Systems	FAB3715A-025
CD69	FN50	Alexa Fluor700	Biolegend	310922

Supplementary Table 3: List of antibodies used for intracellular flow cytometry staining

Antigen	Clone	Fluorochrome	Supplier	Identifier
IL-2	MQ1-17H12	PE	eBioscience	12-7029-71
IL-4	MP4-25D2	PE/Dazzle594	Biolegend	500832
IFN-γ	B27	Alexa Fluor 700	Biolegend	506516
IL-6	MQ2-13A5	Pacific Blue	Biolegend	501114
IL-13	JES10-5A2	PE/Cy7	Biolegend	501914
Granzyme B	QA16A02	APC/Fire750	Biolegend	372210
Granzyme K	GM26E7	FITC	Biolegend	370508

Supplementary Table 4: List of primers used for qPCR

Gene	FWD (5'→ 3')	REV (5'→ 3')
GAPDH	ATCTTCTTTTGCGTCGCCAG	TTCCCCATGGTGTCTGAGC
RPS27a	TGGCTGTCCTGAAATATTATAAGGT	CCCCAGCACCACATTCATCA
TBP	GCTTCGGAGAGTTCTGGGATTG	GCAGCAAACCGCTTGGGATTA
COL1A1	AGATCGAGAACATCCGGAG	AGTACTCTCCACTCTTCCAG
COL3A1	CCTGGAATCTGTGAATCATGCC	TGCGAGTCCTCCTACTGCTA
FN1	CCCAGTCCACAGCTATTCCT	TTCATTGGTCCGGTCTTCTC
FN1EDA	TTCAGACTGCAGTAACCAACAT	GGTCACCCTGTACCTGGAAAC
PLOD2	AAGACTCCCCTACTCCGGAAA	AGCAGTGGATAATAGCCTTCCAA
FAP	GCTTTGAAAAATATCCAGCTGCC	ACCACCATACACTTGAATTAGCA
ACTA2	CTGACCCTGAAGTACCCGATA	GAGTGGTGCCAGATCTTTTCC
PDPN	GGTGCAATCATCGTTGTGGTTA	TTCAGCTCTTTAGGGCGAGTAC
IL6	AGCCCACCGGGAACGA	GGACCGAAGGCGCTTGT
HLA-ABC	TACCTGGAGAACGGGAAGGA	GTGGCCTCATGGTCAGAGA
HLA-DR	CCCTGCAGCACCACAAC	GGAACCACCTGACTTCAATGC
TGFB1	GAGGTCACCCGCGTGCTA	TGCTTGAACTTGTCATAGATTTCGTT
